# Supplementary material for: Antiferromagnetic insulating state in layered nickelates at half filling
Source: Sci Rep. 2022 Oct 25;12:17864. doi: 10.1038/s41598-022-22176-2 (PMC9596485; doi:10.1038/s41598-022-22176-2)
Supplement: Supplementary file 1 — Supplementary Information. [file 41598_2022_22176_MOESM1_ESM.pdf]

Supplemental Material for "Antiferromagnetic insulating state in layered nickelates at half-filling"

Myung-Chul Jung,[1] Harrison LaBollita,[1] Victor Pardo,[2,3] and Antia S. Botana[1]

[1] *Department of Physics, Arizona State University, Tempe, AZ 85287, USA*

[2] *Instituto de Materiais iMATUS, Universidade de Santiago de Compostela, E-15782 Santiago de Compostela, Spain*

[3] *Departamento de Física Aplicada, Universidade de Santiago de Compostela, E-15782 Santiago de Compostela, Spain*

DENSITY OF STATES OF HIGHER-ORDERED NICKELATES

The projected densities of states (PDOS) of La-based n=3-6 reduced nickelates corresponding to the band structures in Fig. 2 of the main text are shown in Fig. 1. For all materials, the d_{z^2} orbitals, which are dominantly located from -2 eV all the way up to the Fermi energy, are fully occupied. The only Ni-d band that remains unoccupied is a single minority-spin $d_{x^2-y^2}$ band per Ni. The oxygen-p states (shown in Fig. 1 in purple color) appear at lower energies. These O-p states shift up in energy with decreasing n.

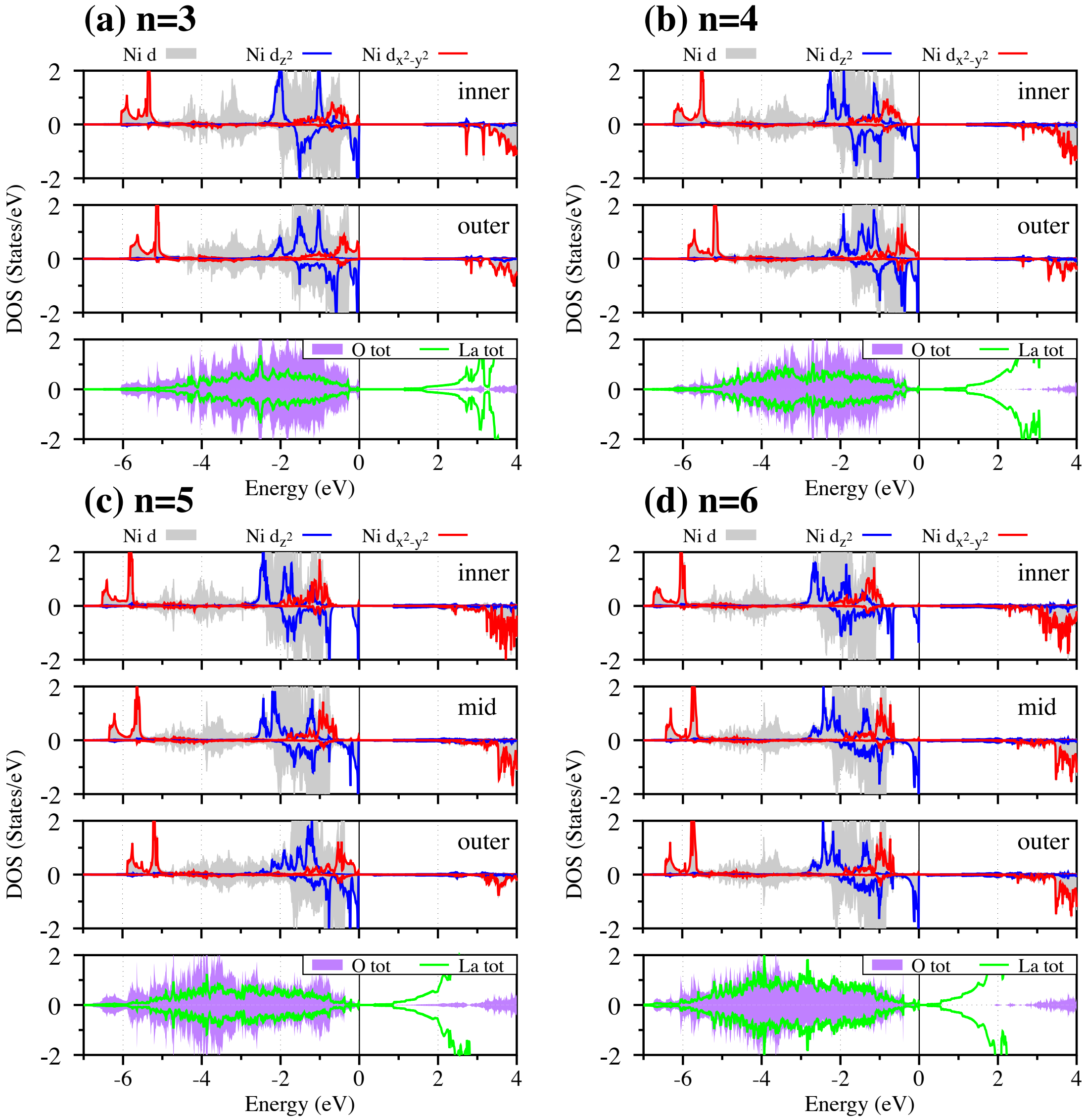

FIG. 1. Orbital-projected density of states of higher-order layered RP phases in GGA+U (U= 5 eV, J= 0.7 eV) at d^9 filling.

FAT BANDS SHOWING THE $d_{x^2-y^2}$ CHARACTER

We provide in Figs. 2 and 3 the fat bands for bulk and monolayer $LaNiO_2$ highlighting the $d_{x^2-y^2}$ orbital contribution, in order to complement the corresponding d_{z^2} bands discussed in the main text. In these plots, we can observe that the self-doping effect in the bulk produces a partial depopulation of the Ni $d_{x^2-y^2}$ band. However, in the monolayer limit, the self-doping effect is no longer present and the $d_{x^2-y^2}$ band is completely half-filled. This latter argument applies to the multilayer system as well.

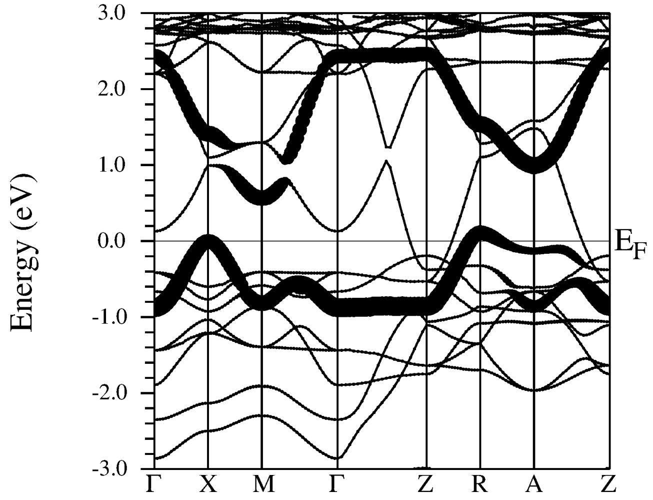

FIG. 2. Band structure of bulk $LaNiO_2$ in C-type AF configuration (in-plane $\sqrt{2} \times \sqrt{2}$ unit cell). The bands highlighted correspond to the $d_{x^2-y^2}$ fat bands coming from the two inequivalent Ni atoms in the structure (exchange split due to the AF coupling). For each spin channel, there is one unoccupied $d_{x^2-y^2}$ band and another one which becomes partly depopulated due to the self-doping effect.

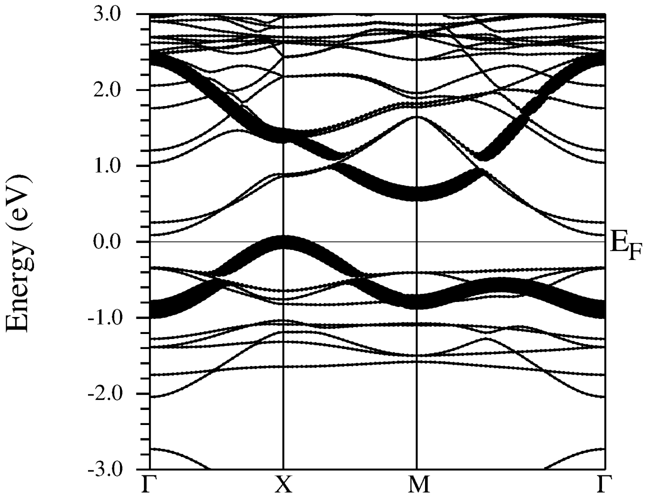

FIG. 3. Band structure of monolayer $LaNiO_2$ in the checkerboard AF configuration (in-plane $\sqrt{2} \times \sqrt{2}$ unit cell). The bands highlighted correspond to the $d_{x^2-y^2}$ fat bands coming from the two inequivalent Ni atoms in the structure (exchange split due to the AF coupling). We see that for each spin channel (only one shown) there is a fully unoccupied $d_{x^2-y^2}$ band and another one that is fully occupied, leading to a fully gapped electronic structure.

NDNIO$_2$ CALCULATIONS

We provide a description of the electronic structure of the $NdNiO_2$ systems analogous to the La-based ones presented in the main text (with the same computational parameters described therein). Figure 4 shows the band structure and DOS of the minority-spin channel of the $NdNiO_2$ monolayer (the Nd moments are considered all parallel so that in the minority-spin channel, the Nd-f bands are completely unoccupied away from the Fermi level). The Ni-d_{z^2} bands are completely occupied and present a small dispersion.

Fig. 5 shows a multilayer system analogous to that in the main text, but using Nd-based materials. Again, the presence of a blocking structure that cuts the c-axis dispersion leads to a gap opening at the Fermi level. In this case, an even smaller U is able to open the gap when compared to the La-based case. Again, the electronic structure is presented for the minority-spin channel only, with the Nd-f bands unoccupied well above the Fermi level.

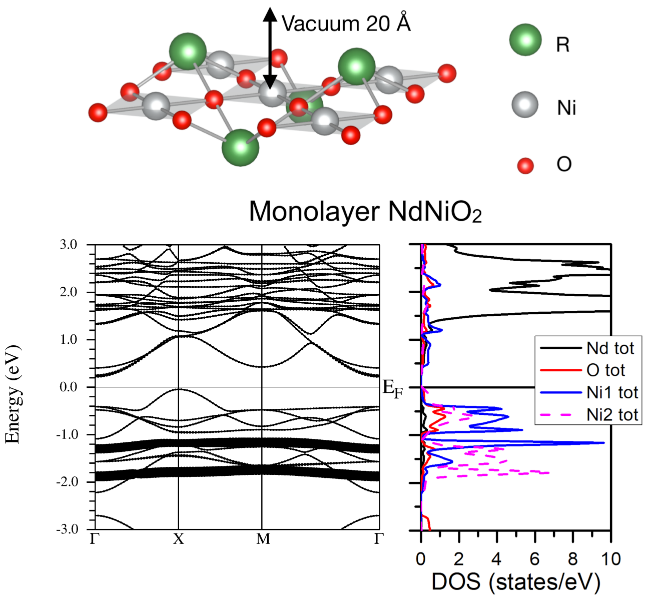

FIG. 4. Top panel. Structure of a $NdNiO_2$ monolayer. Bottom left panel. Band structure of the $NdNiO_2$ monolayer with AF ordering of the Ni atoms, showing only the minority spin channel (Nd moments are parallel). The Ni-d_{z^2} states are highlighted for both Ni atoms (a Hund splitting can again be observed between them). The flat Nd-f bands (unoccupied) appear 2-3 eV above the Fermi level. The O-p bands (not shown) start to appear below -3 eV. A gap opening together with the antiferromagnetic ordering occurs when the off-plane coupling is suppressed. This is visible even at the $U = 0$ limit (bands shown), similar to the situation in bulk $CaCuO_2$, and unlike the self-doped metallic phase appearing in bulk $NdNiO_2$. Bottom right panel. Corresponding Nd, O and Ni atom-resolved density of states (both Ni atoms have opposite spins).

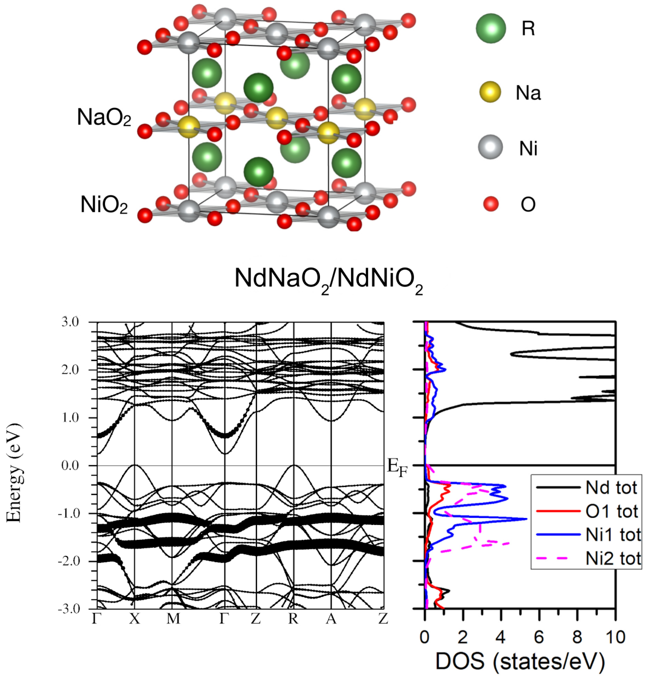

FIG. 5. Top panel. Supercell constructed alternating one layer of $NdNiO_2$ and one layer of $NdNaO_2$. The Ni-Ni direct off-plane hopping is blocked structurally by a single layer of $NdNaO_2$. However, the Nd-d off-plane hopping is not blocked. Na atoms in yellow, R atoms in green, O atoms in red, Ni atoms in gray. Bottom left panel. Band structure of a multilayer $(NdNaO_2)_1/(NdNiO_2)_1$ obtained with a very small U= 0.7 eV. A gap opening together with antiferromagnetic ordering occurs when the off-plane coupling is geometrically suppressed, even when the Nd-d-Nd-d off-plane hopping is permitted. Only the minority spin is shown so that the Nd-f states are unoccupied and away from the Fermi level. The Ni-d_{z^2} bands are highlighted for both Ni atoms antiferromagnetically coupled (a Hund splitting can be noticed between them, as before). Bottom right panel. Corresponding Nd, O (only the O atoms in the NiO_2 plane are shown), and Ni atom-resolved density of states.
